# Membrane Protein Modulators in Neuroinflammation

**DOI:** 10.2174/011570159X375716250701111204

**Published:** 2025-07-17

**Authors:** Ligang Chen, Zheng Zou, Chao Dang, Geyu Wang, Tingzhun Zhu, Guobiao Liang

**Affiliations:** 1 Department of Neurosurgery, General Hospital of Northern Theater Command, Shenyang, Liaoning, 110016, China

**Keywords:** Membrane proteins, modulators, neuroinflammation, therapeutic targets, neurodegenerative diseases, microglia

## Abstract

Neuroinflammation has emerged as a critical pathological process that significantly contributes to the development and progression of a wide range of neurological disorders, including Alzheimer’s disease, Parkinson’s disease, and multiple sclerosis. Recent advances in neuroscience have underscored the pivotal role of neuroinflammation not only in exacerbating these diseases but also in accelerating neuronal degeneration. The growing prevalence of these conditions worldwide, coupled with the limited efficacy of current therapeutic approaches, highlights the urgent need for new therapeutic strategies. Given the central role of neuroinflammation in disease progression, targeting the neuroinflammatory process offers a compelling opportunity for effective intervention. Membrane proteins are key regulators in cellular signal transduction and intercellular communication, and their dysregulation may trigger and sustain neuroinflammatory responses. Consequently, modulators of membrane proteins have emerged as promising candidates for managing neuroinflammation. Current research indicates that natural products and small-molecule compounds can modulate membrane protein activity, effectively mitigating excessive inflammatory responses and exhibiting potent anti-neuroinflammatory effects. This review systematically examines the classification and functional roles of membrane proteins in neuroinflammation, with a particular focus on the therapeutic potential of channel proteins, transporter proteins, and receptor proteins across various neurological conditions. The identification and development of membrane protein modulators present an innovative and urgent avenue for advancing anti-neuroinflammatory therapies, offering potential breakthroughs in treating these prevalent and debilitating diseases.

## 1 INTRODUCTION

 Neuroinflammation is a key pathological process within the central nervous system (CNS), primarily mediated by microglia, astrocytes, and peripheral immune cells. It has been identified as a central factor in the development and progression of neurodegenerative diseases, including Alzheimer’s disease (AD), Parkinson’s disease (PD), and multiple sclerosis (MS) [1-4]. Chronic neuroinflammation contributes to neuronal damage, blood-brain barrier disruption, and synaptic dysfunction, which accelerates disease progression. Given its critical role in disease pathology, targeting neuroinflammation is a promising therapeutic approach.

While considerable progress has been made in understanding the mechanisms of neuroinflammation, significant gaps remain in the development of targeted therapeutic strategies. One major challenge is the complex and dynamic nature of the inflammatory response in the CNS, which involves multiple cellular pathways and molecular interactions. Additionally, the lack of selective targeting and the potential off-target effects of current treatments further complicate the development of effective therapies.

Membrane proteins, which regulate cellular signaling and intercellular communication, have emerged as crucial modulators of neuroinflammation [5, 6]. Dysregulation of these proteins can trigger and sustain neuroinflammatory responses, making them attractive therapeutic targets. However, research into membrane protein modulators is still in its early stages, and most studies focus on their role in peripheral tissues rather than in the CNS [7, 8]. This review aims to fill this gap by systematically examining the classification, functional roles, and therapeutic potential of membrane proteins in neuroinflammation, particularly in the context of neurological diseases.

By focusing on the therapeutic potential of channel proteins, transporter proteins, and receptor proteins, this review provides new insights into the role of membrane protein modulators in managing neuroinflammation. It also highlights the challenges associated with their development and discusses the future directions of research in this field, offering a novel approach for advancing therapies for neurodegenerative diseases and other chronic neuroinflammatory conditions.

We conducted a systematic literature search of English-language articles published between 2010 and 2024 using the PubMed database. Keywords used included “neuroinflammation,” “membrane proteins,” and “modulators.” Studies were included if they addressed the functional role or therapeutic targeting of membrane proteins in neuroinflammatory processes across neurological diseases. Articles were excluded if they were non-peer-reviewed, unrelated to neuroinflammation, or lacked mechanistic relevance. After removing duplicates, titles and abstracts were screened, followed by full-text evaluation. The selected literature was then thematically categorized based on membrane protein types and disease contexts.

## 2 BASIC CLASSIFICATION OF MEMBRANE PROTEINS

Membrane proteins exhibit diverse functions within the cellular membrane and can be broadly categorized based on their roles into channel proteins, carrier proteins, and receptor proteins. These membrane proteins play essential roles in maintaining cellular homeostasis, facilitating signal transduction, and enabling substance transport.

### 2.1 Channel Proteins

Channel proteins are a critical class of membrane proteins in the cell membrane, primarily responsible for the selective transport of ions and small molecules across the cell membrane. This function is essential for maintaining ion homeostasis and membrane potential [9]. Channel proteins can be broadly classified into ion channels and porins. Ion channels are the most prevalent type, selectively permitting specific ions (such as Na^+^, K^+^, Ca^2+^, *etc*.) to cross the cell membrane, thereby regulating intracellular and extracellular ion concentrations and influencing the cell's electrophysiological properties and signal transduction. Activation of calcium channels leads to an increase in intracellular calcium levels, which, in turn, promotes the release of inflammatory mediators. Studies have shown that calcium channels in microglia, such as CaV2.2, play a pivotal role in neuroinflammation, with calcium signaling modulated by these channels impacting the secretion of various inflammatory factors and intercellular communication [10]. Voltage-gated sodium channels play a crucial role in neural signal transmission, rapidly opening and closing in response to changes in membrane potential [11]. Porins, in contrast, are relatively larger channels that permit the free passage of water molecules and small solutes. For instance, aquaporins are specialized for water transport, playing a key role in maintaining cellular water homeostasis [12]. Additionally, the diversity of channel proteins is also evident in their structural and functional variations. For example, specific channel proteins can form heterodimers, enhancing their functional capacity and regulatory potential [13]. Moreover, acid-sensing ion channels (ASICs) have been found to participate in the release of inflammatory mediators, particularly in the context of chronic pain and neurodegenerative diseases. Aberrant activation of ASICs is closely associated with enhanced inflammatory responses [14]. These studies indicate that channel proteins directly participate in the initiation and progression of neuroinflammation by regulating the release of inflammatory mediators.

Channel proteins not only contribute to the release of inflammatory mediators but also play a crucial role in regulating neuronal excitability. Neuronal excitability refers to the cell’s ability to generate action potentials, a process modulated by various ion channels. Research has shown that, under inflammatory conditions, the function of ion channels in neurons may be altered, leading to abnormal excitability. For instance, dysfunction of sodium and calcium channels can result in neuronal hyperexcitability, potentially triggering neurological disorders such as epilepsy [15, 16]. Additionally, potassium channels in microglia have also been shown to play a significant role in modulating neuronal excitability. Studies indicate that microglia indirectly influence the activity of potassium channels in surrounding neurons by releasing inflammatory mediators, thereby regulating their excitability [17, 18]. Thus, channel proteins in neuroinflammation are involved not only in the release of inflammatory mediators but also in modulating neuronal excitability, impacting the overall functionality and health of the nervous system.

### 2.2 Carrier Proteins

Carrier proteins represent another critical class of membrane proteins, primarily responsible for the transmembrane transport of molecules such as neurotransmitters, amino acids, and glucose. They play a vital role in sustaining synaptic transmission and cellular metabolism [19]. Dysfunction of carrier proteins is closely associated with neuroinflammation, and numerous studies have elucidated this connection [20, 21]. For example, glutamate transporter protein (GLT-1, also known as EAAT2) plays a critical role in regulating glutamate levels in the brain, and its dysfunction is associated with various neuroinflammatory diseases [22, 23]. Insufficient GLT-1 function leads to elevated extracellular glutamate levels, which subsequently activate inflammatory responses, increase neuronal excitability, and induce neuronal damage. This process plays a significant role in the pathology of neurodegenerative diseases such as Alzheimer’s and Parkinson’s disease [24-27].

Another carrier protein associated with neuroinflammation is the monoamine transporter, such as the dopamine transporter (DAT) [28, 29]. Under neuroinflammatory conditions, the expression and function of monoamine transporters can be modulated by inflammatory factors. Studies have shown that dysfunction of the DAT disrupts dopamine reuptake in the brain, leading to neuronal damage and further promoting the onset and progression of neuroinflammation [30-32]. In neurological disorders such as multiple sclerosis, DAT dysfunction is considered to be closely associated with exacerbated neuroinflammation and accelerated neurodegenerative processes [33, 34]. Additionally, GABA transporters (GAT) play a significant role in regulating neuroinflammation, particularly in processes related to inhibitory signal transmission in neurons [35-37]. Studies indicate that GAT dysfunction leads to abnormal local GABA levels, thereby impairing the normal inhibitory function of neurons. This effect is particularly pronounced in neuroinflammation-associated disorders such as epilepsy [38-40]. Abnormal expression and altered function of GAT can increase the risk of neuroinflammation and promote neuronal hyperexcitability [41, 42]. The role of these transporter protein dysfunctions in neuroinflammatory diseases underscores the necessity of investigating their mechanisms and regulatory pathways. Such research not only enhances our understanding of the pathological processes of neuroinflammation but also offers new therapeutic targets for treating neuroinflammation-associated disorders.

### 2.3 Receptor Proteins

Receptor proteins are membrane-bound proteins typically located on the cell membrane, where they mediate signal transduction by binding to ligands such as hormones, neurotransmitters, or other cytokines [43]. They are essential in mediating cellular responses to extracellular stimuli and in orchestrating the regulation of intracellular signaling pathways [44]. In neuroinflammation research, various receptor proteins have become focal points due to their critical roles in cellular signal transduction. These receptor proteins participate in the initiation, maintenance, and regulation of neuroinflammation through distinct signaling pathways, profoundly impacting the progression of neurological disorders.

G protein-coupled receptors (GPCRs) are critical targets in the regulation of neuroinflammation. Among them, the adenosine A2A receptor (A2AR), highly expressed in neurons and microglia, promotes the release of pro-inflammatory cytokines (such as TNF-α and IL-6) *via* the cAMP-PKA signaling pathway [45-47]. In neurodegenerative diseases such as Alzheimer’s and Parkinson’s, A2AR activation is closely associated with exacerbated inflammation [48, 49]. Another important GPCR is the chemokine receptor CXCR4, which, upon binding to its ligand CXCL12, activates the PI3K/Akt and ERK pathways, thereby promoting neuroinflammation [50]. CXCR4 is highly expressed in inflammatory diseases such as multiple sclerosis, and inhibiting its activity can attenuate neuroinflammatory responses [51-53]. The active involvement of CXC chemokine receptor 2 (CXCR2) in brain trauma and stroke highlights its role in neuroinflammation [54-58]. CXCR2 promotes inflammatory responses through the NF-κB pathway, and its inhibitors have shown potential in alleviating neuroinflammatory damage [59, 60]. Additionally, the histamine H4 receptor (H4R) induces pro-inflammatory responses in microglia by inhibiting cAMP and activating the MAPK pathway [61-63]. Experimental studies have shown that H4R antagonists can significantly reduce neuroinflammation in disease models of Parkinson’s disease, neuropathic pain, and amyotrophic lateral sclerosis [61, 64-66]. The role of angiotensin receptors is particularly prominent in neurological disorders such as Parkinson’s disease. Their activation of the MAPK and NF-κB pathways promotes pro-inflammatory responses in microglia. Modulating angiotensin activity can effectively mitigate neuroinflammation [67, 68].

Tumor necrosis factor receptors (TNFR), particularly TNFR1, initiate NF-κB and MAPK signaling pathways in inflammatory responses by binding to TNF-α, thereby inducing the production of numerous pro-inflammatory cytokines [69, 70]. TNFR1 is highly expressed in Alzheimer’s and Parkinson’s diseases, further exacerbating the chronic inflammatory environment and contributing to neuronal damage [71, 72]. Inhibition of TNFR has been investigated as a means to reduce neuroinflammation and slow disease progression [73-75]. Additionally, the N-methyl-D-aspartate receptor (NMDA receptor), as a crucial ion channel receptor, plays a key role in the regulation of neuronal excitability [76]. Excessive activation of NMDA receptors leads to an influx of calcium ions, triggering oxidative stress and inflammatory responses, ultimately resulting in neuronal damage [77]. NMDA receptor antagonists have demonstrated neuroprotective effects in ischemic brain injury and multiple sclerosis [78, 79]. Peroxisome proliferator-activated receptors (PPARs), particularly PPARγ, have garnered attention for their anti-inflammatory properties. PPARγ reduces levels of inflammatory markers by inhibiting the expression of pro-inflammatory genes, demonstrating significant neuroprotective effects in models of Alzheimer’s and Parkinson’s diseases [80, 81]. Scavenger receptor class A (SR-A), primarily expressed on microglia, recognizes and clears danger signals and pathological proteins. However, excessive activation of SR-A can also release pro-inflammatory cytokines, exacerbating the inflammatory environment [82]. Although SR-A contributes to the clearance of pathological proteins, such as β-amyloid in Alzheimer’s disease, its pro-inflammatory effects may inadvertently accelerate disease progression. The complex roles of these receptor proteins in neuroinflammation underscore their critical significance in neurological disorders. By modulating the activity of these receptors, particularly targets like GPCR, TNFR, and NMDA receptors, it may be possible to develop effective anti-neuroinflammatory therapies. This approach not only aids in elucidating the pathological processes of neuroinflammation but also opens new avenues for treating neurodegenerative diseases.

 Membrane proteins play an essential role in cellular signal transduction and intercellular communication, particularly in the regulation of neuroinflammation. These proteins include ion channels, transporters, and receptors, each of which contributes to the activation, propagation, and resolution of neuroinflammatory responses in the CNS. Ion channels, such as voltage-gated sodium and potassium channels, facilitate the influx and efflux of ions, thus contributing to neuronal excitability and inflammation. Transporter proteins, including glutamate and serotonin transporters, regulate the levels of neurotransmitters and other signaling molecules, which are critical in maintaining neuroinflammation balance. Receptor proteins, such as G-protein-coupled receptors (GPCRs) and cytokine receptors, mediate the cellular response to external signals, influencing inflammatory pathways and neuronal survival.

## 3 RESEARCH ON MEMBRANE PROTEIN MODULATORS IN DIFFERENT DISEASES

Membrane protein modulators have shown potential in treating various neurological disorders by targeting key pathways of neuroinflammation, thereby providing a critical foundation for the development of therapeutic strategies (Table **1**).

### 3.1 Multiple Sclerosis

Multiple sclerosis is an autoimmune disease of the central nervous system with diverse and complex clinical manifestations, including motor impairments, sensory abnormalities, visual issues, and cognitive dysfunction. Membrane protein modulators have gained attention as an emerging therapeutic approach. By regulating the functions of proteins on the cell membrane, these agents may offer new directions and hope for the treatment of MS.

Kv1.3 potassium channel blockers, such as margatoxin and 4-aminopyridine, reduce autoimmune responses in MS by inhibiting potassium efflux, thereby decreasing T cell activation [83-87]. Sodium channel blockers, such as lamotrigine, support neuronal stability and reduce excitotoxicity by inhibiting voltage-gated sodium channels, particularly Nav1.6 [88, 89]. Additionally, GLT-1 activators, such as ceftriaxone, play a key role in clearing glutamate from the synaptic cleft and suppressing associated glial activation [90]. TLR2 and TLR4 are important targets for membrane protein modulators in MS, where inhibiting their expression can reduce pro-inflammatory responses in microglia, thereby slowing disease progression [91]. Additionally, EAAT2 enhancers, such as riluzole, exert neuroprotective effects by modulating voltage-gated sodium channels and glutamate transport. PPARγ agonists, including rosiglitazone and pioglitazone, inhibit microglial activation and reduce the release of pro-inflammatory cytokines, thereby slowing the progression of MS [92, 93]. NMDA receptor antagonists, such as memantine and amantadine, can mitigate neurological symptoms of the disease and reduce the expression of pro-inflammatory cytokines in the brain [78].

### 3.2 Neurodegenerative Diseases

Neurodegenerative diseases are characterized by the gradual loss and functional decline of neurons and primarily include Alzheimer’s disease, Parkinson’s disease, and Huntington’s disease. Abnormal structure and function of membrane proteins are closely associated with various neurodegenerative diseases. For instance, the aggregation of amyloid-β peptides in Alzheimer’s disease is significantly linked to changes in membrane lipid composition [94]. In-depth research into the structure and function of membrane proteins can enhance our understanding of the pathogenesis and progression of these diseases, offering avenues for effective interventions. Moreover, studies on membrane proteins provide new perspectives for the discovery of biomarkers and clinical diagnostics, potentially aiding in the early identification and monitoring of neurodegenerative disease progression [95]. Therefore, the importance of membrane proteins in neurodegenerative disease research cannot be overstated. In the pathogenesis of AD, the accumulation of β-amyloid (Aβ) is a prominent pathological hallmark. The membrane protein processing of amyloid precursor protein (APP) plays a critical role in Aβ generation. Endogenous cleavage of APP is primarily mediated by γ-secretase and β-secretase, with the activity of the latter regulated by multiple membrane proteins. For instance, studies have shown that presenilin, as the core component of γ-secretase, participates in APP processing, and its dysfunction is closely associated with the onset of AD [96]. Membrane fluidity and composition can also impact the trafficking and cleavage of APP, thereby influencing Aβ production. Recent studies suggest that age-related changes may affect neuronal transport of APP, leading to abnormal Aβ accumulation [97]. Understanding the role of membrane proteins in APP processing is therefore crucial for elucidating the pathological mechanisms of AD. Additionally, in Alzheimer’s disease, the Caspase-1/IL-1β pathway inhibits the membrane trafficking of GluA1 by disrupting the interaction between the membrane protein Stargazin and GluA1, resulting in synaptic damage [98]. Receptor for Advanced Glycation End-products (RAGE) inhibitors, such as FPS-ZM1, have been investigated as potential treatments for AD by reducing Aβ-induced inflammation [99, 100]. DMXBA (GTS-21), as an agonist of the α7 nicotinic acetylcholine receptor (α7nAChR), has been shown to reduce inflammatory responses, providing evidence for neuroprotection in neurodegenerative diseases [101, 102]. Studies on Alzheimer’s disease patients have found that rosiglitazone can significantly improve cognitive function and reduce markers of brain inflammation, indicating its potential in anti-neuroinflammatory therapy [103-105].

DAT plays a crucial role in dopamine reuptake, and its dysfunction can lead to excessive dopamine loss, resulting in motor impairments and other neuropsychiatric symptoms [106]. In PD, neuroinflammation contributes to the degeneration of dopaminergic neurons, and several membrane protein modulators have demonstrated potential therapeutic benefits. P2X7 receptor antagonists, such as AZ10606120 and JNJ-47965567, protect neurons from degeneration by reducing microglial activation and the release of pro-inflammatory cytokines [107, 108]. The activation of A2AR in microglia typically increases the production of pro-inflammatory cytokines, leading to an inflammatory response. Adenosine A2A receptor (A2AR) antagonist istradefylline effectively reduces the production of inflammatory markers by inhibiting A2AR activity, thereby improving motor symptoms in PD patients [109, 110]. TRPM2 is a calcium channel highly sensitive to calcium influx under oxidative stress conditions in neuronal cells. In Parkinson’s disease models, TRPM2 inhibitors, such as AG490, reduce neuroinflammation and oxidative stress, thereby preventing neuronal degeneration and glial cell morphological changes [111-113].

### 3.3 Traumatic Brain Injury

Traumatic brain injury (TBI) can result in acute and chronic neural damage, leading to cognitive, emotional, and behavioral impairments that severely impact patients' quality of life. Membrane proteins play a crucial role in these pathological processes by participating in cell signaling, ion channel regulation, and intercellular interactions, among other vital physiological functions. Calcium channel blockers, such as nimodipine and verapamil, help reduce neuronal calcium influx, which in turn reduces inflammation and improves prognosis in TBI patients [114-116]. P2X7 receptor antagonists exhibit significant neuroprotective potential in TBI, particularly by suppressing pro-inflammatory pathways and alleviating TBI-induced neuroinflammation [117, 118]. Aquaporin-4 (AQP4) is a water channel protein primarily expressed in the CNS. In TBI, AQP4 expression is abnormally upregulated, leading to excessive water influx into brain tissue and resulting in cerebral edema. Minocycline mitigates post-TBI cerebral edema by inhibiting AQP4 and enhances the recovery of neurological function [119, 120]. A2AR antagonists, such as istradefylline, have shown potential not only in PD but also in TBI by reducing neuroinflammation and promoting functional recovery [121].

### 3.4 Spinal Cord Injury

CX3CL1 is a chemokine expressed by neurons that can exist in either membrane-bound or soluble forms. Tetrodotoxin alleviates spinal cord injury (SCI) and promotes neurological recovery by inhibiting sodium channels [122, 123]. After SCI, CX3CL1 expression increases, promoting the recruitment and activation of microglia, which triggers further pro-inflammatory responses. Inhibiting CX3CR1 can reduce excessive microglial activation, thereby alleviating secondary damage and ultimately improving functional recovery following SCI [124]. CSF1R inhibitors, such as GW2580 and PLX5622, have demonstrated efficacy in SCI treatment by reducing inflammation and apoptosis [125, 126].

### CONCLUSION AND FUTURE RESEARCH DIRECTIONS AND CHALLENGES

In models of neuroinflammation, membrane protein modulators have demonstrated promising therapeutic potential; however, their precise mechanisms of action within cellular pathways remain insufficiently understood. Pathways such as NF-κB and MAPK play essential roles in initiating and sustaining neuroinflammatory responses, yet the specific modulatory effects of these agents on these pathways are still to be elucidated. To address this gap, future research should comprehensively explore these regulatory mechanisms across various cellular and animal models, aiming to substantiate the modulators’ anti-inflammatory effects with robust empirical evidence.

Another critical focus of future studies is to systematically evaluate the long-term safety and efficacy of membrane protein modulators in human populations. The rigorous assessment of these agents’ toxicity, tolerance, and pharmacokinetic properties will be essential to ensuring their safe application in the clinical treatment of neurological disorders. Given the heterogeneity of neurodegenerative diseases, clinical trials targeting specific disease types and stages will provide the data needed to optimize the therapeutic potential of membrane protein modulators.

The integration of membrane protein modulators with existing treatment regimens also presents a promising avenue for enhancing therapeutic efficacy. Investigating the combined effects of membrane protein modulators with anti-inflammatory agents, neuroprotective drugs, and immunomodulators could reveal synergistic benefits. This approach may be especially valuable for neurodegenerative conditions such as Alzheimer’s and Parkinson’s, where combined treatments could more effectively mitigate neuroinflammation and slow disease progression. For instance, Commiphora myrrh has demonstrated promising anti-inflammatory and neuroprotective effects, highlighting its potential as a modulator of neuroinflammation [127]. Similarly, sitagliptin, a DPP4 inhibitor, has shown beneficial effects in reducing inflammatory markers in non-diabetic COVID-19 patients, pointing to its broader application in managing neuroinflammation [128]. Furthermore, the use of glibenclamide in mitigating neuroinflammation through the inhibition of NLRP3 inflammasomes and oxidative stress further supports the therapeutic promise of targeting membrane proteins in neuroinflammatory diseases [129]. The modulation of brain-derived neurotrophic factor (BDNF) signaling also offers a promising avenue for neuroprotection, especially in diseases like multiple sclerosis [130].

The identification and development of membrane protein modulators provide an innovative approach to treating neuroinflammation. Continued research into these modulators will be essential in advancing therapies for neurodegenerative diseases and other conditions associated with chronic neuroinflammation. To improve the specificity and targeting capacity of membrane protein modulators, structural optimization will be a key research priority. Advanced techniques such as molecular docking and computational modeling can help refine the binding affinity of modulators to specific targets, thereby enhancing drug selectivity and therapeutic outcomes. Additionally, targeted delivery systems, such as nanocarriers, hold significant promise for increasing modulatory efficacy while minimizing systemic side effects, potentially opening new avenues for precise and personalized neuroinflammation therapy. Membrane protein modulators exhibit significant therapeutic potential, effectively regulating signaling pathways involved in neuroinflammation and consequently alleviating the pathological progression of neurodegenerative diseases. Current research has demonstrated that natural products and small-molecule compounds can significantly reduce excessive inflammatory responses by modulating membrane protein activity, showcasing promising anti-neuroinflammatory effects. However, there are certain limitations and potential risks associated with translating these modulators into clinical applications. Firstly, the complexity and heterogeneity of membrane protein structures pose challenges for achieving selective targeting, potentially leading to unintended off-target effects and subsequent adverse reactions. Additionally, the widespread distribution of membrane proteins throughout central and peripheral tissues raises the risk of drug-related toxicities, such as nonspecific immunosuppression or metabolic disturbances. Moreover, limitations in drug permeability across the blood-brain barrier (BBB) may reduce effective concentrations within the CNS, further constraining therapeutic efficacy. Therefore, future research should focus on enhancing the selectivity and specificity of membrane protein modulators, developing more precise drug delivery systems, and minimizing potential adverse effects. While these challenges complicate the development of membrane protein modulators, they also clearly define directions for further research. A comprehensive understanding and resolution of these issues will be critical for the successful clinical application of membrane protein modulators.

Membrane protein modulators exhibit significant promise in treating neuroinflammation-related disorders, but further exploration is required to address critical challenges in their clinical translation. Recent clinical trials and advancements in drug development have brought new opportunities to this field. For instance, ongoing clinical studies include antagonists targeting the Transient Receptor Potential Vanilloid type 1 (TRPV1) channel for treating neuropathic pain and neuroinflammation [131, 132], and small-molecule inhibitors selectively targeting the P2X7 receptor are being explored for depression. Additionally, medications such as glibenclamide and sitagliptin have demonstrated promising potential in modulating membrane proteins and alleviating neuroinflammation, thereby being increasingly considered for integration into existing treatment regimens [133, 134]. These modulators could synergize with traditional anti-inflammatory drugs, immunomodulators, and neuroprotective agents, potentially slowing disease progression and enhancing therapeutic efficacy more effectively. Key future research directions include determining optimal combinational therapeutic strategies involving membrane protein modulators, improving drug delivery techniques to ensure efficient penetration across the blood-brain barrier (BBB), and conducting precise clinical trials to validate the long-term safety and efficacy of these combination approaches. Although these tasks present significant challenges, they offer a clear developmental path for researchers, ultimately driving membrane protein modulators from fundamental research toward practical clinical application.

Although this review systematically summarizes the classification, functional mechanisms, and therapeutic potential of membrane proteins in neuroinflammation, several limitations should be acknowledged. First, the literature included was primarily selected from English-language publications over the past decade, which may introduce selection bias and may not encompass all high-quality and recent studies. Second, most current research on membrane protein modulators remains at the stage of animal models or *in vitro* experiments, with a lack of large-scale clinical validation, thus limiting the generalizability and clinical relevance of the conclusions. In addition, inconsistencies in classification criteria and mechanistic interpretations of membrane proteins across studies may affect the overall consistency and systematic nature of this review. It is also worth noting that although this review covers various neurodegenerative diseases, it does not deeply explore the disease-specific roles of membrane proteins, which may limit its applicability to individualized disease contexts. Finally, challenges related to drug delivery, target specificity, and safety of membrane protein modulators in the central nervous system remain unresolved, and further investigation is required for their clinical translation. 

## Figures and Tables

**Table 1:** Summary of membrane protein modulators in neuroinflammation.

**Membrane Protein Modulator**	**Disease**	**Key Findings**	**References**
Kv1.3 Potassium Channel Blocker	MS	Inhibits potassium efflux, reduces T cell activation, and mitigates autoimmune response.	[83-87]
GLT-1 Activator	AD, PD	Regulates glutamate levels, reduces neuroinflammation, and decreases neuronal damage.	[90-92]
A2A Receptor Antagonist	AD, PD, TBI	Reduces pro-inflammatory cytokines by inhibiting A2AR activity, improving motor symptoms, and reducing inflammation.	[109-121]
NMDA Receptor Antagonist	AD, MS	Inhibits NMDA receptor activity, reduces neuronal excitability and oxidative stress, showing neuroprotective effects.	[76-78]
P2X7 Receptor Antagonist	PD, TBI	Reduces microglial activation and pro-inflammatory cytokine release, protecting neurons from degeneration.	[107-118]
TRPM2 Calcium Channel Inhibitor	PD	Decreases neuroinflammation and oxidative stress, preventing neuronal degeneration and glial morphological changes.	[111-113]
Aquaporin-4 Inhibitor	TBI	Reduces AQP4 expression, alleviates brain edema, and improves neurological recovery.	[119-121]
TLR2/TLR4 Inhibitor	MS	Suppresses pro-inflammatory responses in microglia, slowing disease progression.	[91]
CXCR4 Antagonist	MS	Reduces pro-inflammatory cytokine expression by inhibiting CXCR4 activity, alleviating neuroinflammation.	[50-53]
Histamine H4 Receptor Antagonist	PD	Reduces MAPK pathway activity in microglia, showing neuroprotective and anti-inflammatory effects.	[61-66]

## LIST OF ABBREVIATIONS

AD: Alzheimer’s Disease
APP: Amyloid Precursor Protein
BBB: Blood-brain Barrier
BDNF: Brain-derived Neurotrophic Factor
CNS: Central Nervous System
DAT: Dopamine Transporter
GPCRs: G Protein-coupled Receptors
MS: Multiple Sclerosis
PD: Parkinson’s Disease
PPARs: Peroxisome Proliferator-activated Receptors
TBI: Traumatic Brain Injury
TNFR: Tumor Necrosis Factor Receptors
